# Improvement of reverse sequence algorithm for syphilis diagnosis using optimal treponemal screening assay signal-to-cutoff ratio

**DOI:** 10.1371/journal.pone.0204001

**Published:** 2018-09-13

**Authors:** Bouchra Serhir, Annie-Claude Labbé, Florence Doualla-Bell, Marc Simard, Gilles Lambert, Annick Trudelle, Jean Longtin, Cécile Tremblay, Claude Fortin

**Affiliations:** 1 Laboratoire de santé publique du Québec/Institut national de santé publique du Québec, Sainte-Anne-de-Bellevue, Québec, Canada; 2 CIUSSS de l’Est-de-l’Île-de-Montréal, Hôpital Maisonneuve-Rosemont, Service de Maladies infectieuses et microbiologie médicale, Montréal, Canada; 3 Unité de surveillance des maladies chroniques et de leur déterminants/Institut national de santé publique du Québec, Québec, Québec, Canada; 4 Unité des infections transmissibles sexuellement et par le sang/Institut national de santé publique du Québec, Montréal, Québec, Canada; 5 Centre hospitalier de l’Université de Montréal, Département de microbiologie médicale et infectiologie, Montréal, Québec, Canada; Jiangsu provincial Center for Disease Control and Prevention, CHINA

## Abstract

**Background:**

Although reverse sequence algorithms (RSA) for syphilis screening are performing well, they still have to rely on treponemal confirmatory tests at least for sera reactive by enzyme immunoassay/chemiluminescence immunoassay (EIA/CIA) and unreactive by rapid plasma reagin (RPR). Quebec’s laboratory network previously showed that 3.3% of EIA/CIA reactive and weakly-reactive RPR samples (RPR titer of 1 to 4) would have been misclassified as syphilis cases if a treponemal confirmatory test had not been performed.

**Objectives:**

To correlate the magnitude of signal-to-cutoff (S/CO) ratios of the 4 most used commercial first-line EIA/CIA kits in Quebec with syphilis confirmation results and establish a S/CO value above which treponemal confirmation would not be required.

**Methods:**

Serum samples from previously undiagnosed individuals (n = 7 404) obtained between January 2014 and February 2017 that were reactive by EIA/CIA and either negative by RPR or reactive with a low titer (1 to 4) were included in the study. All samples were tested with *Treponema pallidum* particle agglutination (TP-PA) and, if negative or inconclusive, with a line immunoassay (LIA). Syphilis infection confirmation was defined by a reactive TP-PA or LIA. Logistic regression analysis was used to determine S/CO values (95% CI lower bound = 0.98) above which confirmation would not be required. The four kits studied were Architect TP, BioPlex IgG, Syphilis EIA II, and Trep-Sure.

**Results:**

Of 2609 reactive EIA/CIA specimens tested for the determination of S/CO values, 1730 (66%) were confirmed as true syphilis cases. Confirmation rate was significantly higher in samples with low-titer positive RPR (92%) than with negative RPR samples (54%); p<0.01. A linear probability model (95% CI lower bound = 0.98) predicted the S/CO value above which a confirmation would no longer be needed for the Architect TP (16.4), Bioplex IgG (7.4) and Trep-Sure (24.6). No linearity was observed between the S/CO value of Syphilis EIA II and the confirmation rate. The validity of the predicted S/CO values was investigated using 4 795 specimens. The use of an S/CO value of 16.4 with the Architect TP kit and of 24.6 for the Trep-Sure kit would obviate the need for confirmation of 18.5% and 13.2% of sera from the all RPR subgroup, respectively. For the BioPlex IgG kit, 81.1% of sera would not require confirmation when using the S/CO value of 7.4 in the low titer RPR subgroup.

**Conclusion:**

Signal-to-cut-off values could be used to identify sera that do not require extra treponemal confirmation for 3 of the 4 most used first-line EIA/CIA kits in Quebec. Using these values in our current reverse screening algorithm (RSA) would avoid the need for confirmatory tests in 14 to 20% of sera, a proportion that could reach 75% among low-titer RPR.

## Introduction

Syphilis is an infectious disease caused by *Treponema pallidum*. With only 3 cases reported in Quebec in 1998 (0.04 per 100,000 people), it was possible to hope that syphilis could be eradicated. However, since early 2000s, the resurgence of this infection in Quebec resulted in incidence rates reaching 7.1 per 100,000 people in 2015 [1].

The traditional algorithm of syphilis serodiagnosis starts with a subjective manual assay using a non treponemal test (NTT), the rapid plasma reagin (RPR). This NTT, which detects non-specific anticardiolipin antibodies, is followed, when reactive, by a treponemal test (TT) that detects anti-treponemal antibodies for confirmation [1,2].

In Quebec, serodiagnosis of syphilis is performed by 80 medical laboratories, each serving its associated territory [3]. The recrudescence of the disease in high-prevalence areas led laboratories to implement more efficient screening testing notwithstanding the increasing volume of tests and the associated workload. In 2009, several laboratories in Quebec adopted a reverse screening algorithm (RSA) in which a TT (EIA or CIA), which can be automated, is first performed followed by a reflex RPR when positive [4]. A similar algorithm was acknowledged by the CDC in 2008 [5].

Over 25 medical laboratories (representing 80% of testing volume) use a RSA in Quebec. Before 2013, patient’s specimens negative by EIA/CIA are reported as syphilis negative; specimens positive by EIA/CIA and positive by RPR were reported as syphilis positive while those with positive EIA/CIA and negative RPR were sent to the provincial reference laboratory, the Laboratoire de santé publique du Québec (LSPQ), for confirmation. This algorithm was modified in 2013 after an evaluation of the confirmation rate of specimens positive by EIA and weakly positive by RPR was achieved. In fact, a total of 427 patients samples collected between December 2011 and February 2013 presenting with positive EIA/CIA (BioPlex 2200 Syphilis IgG and Syphilis EIA-II from Bio-Rad Laboratories; Architect Syphilis TP from Abbott Laboratories, and Captia Syphilis TA and Trep-Sure Syphilis Total Antibody EIA from Trinity BioTech) and positive RPR (Macro-Vue RPR Card Test from Becton-Dickinson, RPR Carbon Antigen Test from Pulse Scientific, and Wampole Impact RPR Test kit from Alere) with low titer (titer of 1, 2, 4 or 8) were tested by TP-PA and/or Inno-Lia immunoassay (LIA). Of the 427 patients tested, 97% were confirmed positive (92% for RPR titer 1, 98% for RPR titer 2, 99% for RPR titer 4 and 100% for RPR titer 8). The 14 remaining patients were confirmed negative with both TP-PA and LIA and de facto misclassified as syphilis cases [6]. These data led us to perform confirmatory TT on all sera positive by EIA/CIA and a RPR that are either negative or positive at a titer of 1, 2 or 4.

Recent studies demonstrated some correlation between EIA/CIA signal-to-cutoff (S/CO) ratio’s values and true syphilis infection status as determined by confirmatory testing [7–10]. Our study aimed to evaluate the correlation between the S/CO values and the confirmation rate for the four most used syphilis screening kits in Quebec, then to establish and validate a signal strength value associated with a confirmation rate of 100%. This study could allow us to come up with a more efficient and eventually cost effective RSA.

## Materials and methods

### Patients and specimens

We compiled the available data from a total of 7 404 clinical serum samples collected from patients previously undiagnosed with syphilis and submitted for syphilis confirmation at the LSPQ. No laboratory analysis was performed for this project. The samples included in this study were collected from patients with no previous confirmed syphilis serology in Quebec. Samples were not submitted to many freeze and thaw cycles. The data analysed were collected as part of the Quebec routine diagnostic procedures and did not require written informed consent due to their retrospective nature and the fact that information obtained would not be linked to identifying information. All sera were reactive using a TT, and were either non-reactive or reactive with a low titer (1 to 4) by RPR.

Amongst the 7 404 samples, 2 609 submitted between January 2014 and April 2015 were used to established the S/CO values for each of the studied EIA/CIA kits, while 4 795 submitted between May 2015 and February 2017 were used to validate the previously predicted S/CO value for each kit.

### First-line assays

Twenty-three laboratories in Quebec participated to this study. Four recombinant antigens-based TT were used: a chemiluminescence immunoassays (Architect Syphilis TP assay; Abbott Laboratories) two enzyme immunoassays (Syphilis EIA II Total Antibody assay; Bio-Rad Laboratories, Trep-Sure Syphilis Total Antibody EIA; Trinity BioTech), and a multiplex flow immunoassay (Bioplex 2200 Syphilis IgG; Bio-Rad Laboratories). Three nontreponemal test were also used in this study: Macro-Vue RPR Card Test from Becton-Dickinson, RPR Carbon Antigen Test from Pulse Scientific and Wampole Impact RPR Test kit from Alere.

All TT and NTT were performed according to the manufacturer’s recommendations. Architect TP, Bioplex IgG and Syphilis EIA-II kits express the results as a S/CO index with a cut-off at 1.00. Trep-Sure expresses the results as an optical density (OD) with a variable cut-off. In order to standardize the expression of all results, the Trep-Sure OD results were converted into S/CO ratios with a cut-off of 1.00.

All results were expressed as normalized S/CO ratios obtained by measuring the signal strength of sample and the signal strength of an internal cutoff. Samples with an S/CO ratio of ≥1.0 are defined as positive according to the manufacturers.

### Confirmation assays

All sample were further tested with TP-PA (Fujirebio Diagnostics, Inc., Seguin, Texas) followed by InnoLia (Fujirebio Europe, N.V., Gent, Belgium) if the TP-PA was negative or inconclusive [11]. All confirmatory TT were performed according to the manufacturer’s instructions. Finally, samples positive with either TP-PA or InnoLia were considered positive for syphilis and those negative or indeterminate with InnoLia were considered as negative and were not included in this study. Sera presenting with reactive screening TT and reactive NTT at a titer of ≥8 were not submitted for TP-PA and/or Inno-Lia confirmation as they are considered true syphilis cases neither they were included in the study.

### Statistical analysis

Analysis of categorical variables was performed using a Chi-square test, p <0.001. In order to take into account the random variability influenced by the number of tests performed using each commercial assays, we determined the S/CO confirmatory values as correlating with 100% confirmation rate by modelling predicted syphilis confirmation rate for each assays. Predicted syphilis confirmation rate for each screening assay were estimated using logistic regression models when minimal samples size were met (10 events by model) [12]. The S/CO confirmatory value was defined as the value for which the predicted value lower bound of the 95% confidence interval (CI) is equal 0.98. In order to take into account the sampling variability, predicted confirmation rate lower bound CI was favored over predicted confirmation rate value. The validity of logistic regression models to predict syphilis confirmation rate were assessed using model performance measures. Indeed, discrimination, defined as the ability to assess which test confirmed syphilis infection, was quantified by the area under the receiver operating characteristic curve (AUC), [13]. We considered an AUC<0.70 to represent poor discrimination, 0.70–0.79 acceptable discrimination, 0.80–0.89 excellent discrimination and ≥0.90 outstanding discrimination [14]. We graphically assessed calibration (i.e., the agreement between observed outcome and the model’s predicted probabilities [13], by comparing agreement between observed syphilis confirmation rate and predicted confirmation rate. Statistical tests were two-sided with significance assigned at p < 0.05 and analysis were performed using SAS 9.4 (SAS Institute, Cary, NC).

## Results

### Determination of S/CO values to avoid unnecessary confirmation

Confirmation rates of EIA/CIA positive and either negative or weakly reactive RPR samples according to each commercial kit are shown in Table 1.

**Table 1:** Confirmation rates by TP-PA and LIA of samples repeatedly reactive by EIA/CIAscreening tests.

EIA/CIA assays*	All samples		RPR negative samples		Low titer RPR samples				
	*Total*, *n*	*Confirmed samples*, *n (%)*	*Total*, *n*	*Confirmed samples n (%)*	*Total*, *n*	*Confirmed samples*,			
						RPR titer 1 n/total (%)	RPR titer 2 n/total (%)	RPR titer 4 n/total (%)	All low titer RPR *n (%)*
**Architect TP**	668	372 (56%)	441	174 (39%)	227	72/91 (79%)	59/66 (89%)	67/70 (96%)	198 (87%)
**Bioplex IgG**	1358	1004 (74%)	916	581 (63%)	442	166/179 (93%)	157/159 (99%)	100/104 (96%)	423 (96%)
**Syphilis EIA II**	290	177 (61%)	198	101 (51%)	92	28/42 (67%)	32/33 (97%)	16/17 (94%)	76 (83%)
**Trep-Sure**	293	177 (60%)	227	116 (51%)	66	20/24 (83%)	27/28 (96%)	14/14 (100%)	61 (92%)
**Total**	2609	1730 (66%)	1782	972 (55%)	827	286/336 (85%)	275/286 (96%)	197/205 (96%)	758 (92%)

*All threshold S/CO ratios were standardized (S/CO = 1.00).

EIA: enzyme immunoassay; CIA: chemiluminescence immunoassay; RPR: rapid plasma reagin; TP-PA: Treponema pallidum particule agglutination; LIA (Inno-Lia): line immunoassay. Low titer RPR = 1, 2 and 4; Confirmed samples = samples first repeatedly reactive by EIA/CIA then confirmed by TP-PA or LIA

A total of 2 609 clinical samples, on a per-patient basis, were included in the analysis to establish S/CO values. All samples were tested with an NT RPR, 1782 were found RPR negative, 336 RPR reactive at titer 1, 286 RPR reactive at titer 2, and 205 RPR reactive at titer 4. The confirmation rate was 85% (67–93%) for specimens with RPR titer 1, 96% (89–99%) for RPR titer 2 and 96% (94–100%) for RPR titer 4.

From the 2609 samples tested, 1358, 668, 293, and 290 were positive using BioPlex IgG, Architect TP, Trep-Sure, and Syphilis EIA II, respectively. Results were stratified into three categories: negative RPR sera, low-titer RPR sera (1 to 4) and all sera. Of the 2609 serum samples tested, 1730 (66%) were confirmed as true syphilis cases. The confirmation rate was significantly higher (p<0.001) in samples with low-titer RPR (89.5%) as compared to samples with negative RPR (51.2%), independently of the screening test used.

A correlation between the observed S/CO values and confirmation rate was observed with the Architect and Trep-Sure kits for all sera and the for two RPR subgroups, Table 2.

**Table 2:** Determination of observed and predicted S/CO values above which no confirmatory testing would be required, January 2014-April 2015.

EIA/CIA Assays (number of laboratories)		All samples			RPR negative samples			Low titer RPR samples		
		*Total*, *n*	*S/CO*	*Unrequired sample confirmation*, *n (%)*	*Total*, *n*	*S/CO*	*Unrequired sample confirmation*, *n (%)*	*Total*, *n*	*S/CO*	*Unrequired sample confirmation*, *n (%)*
**Architect TP** (13)	Observed S/CO	668	16.1	137 (20.5%)	441	16.5	31 (7.0%)	227	6.9	161 (70.9%)
	Predicted S/CO		16.4	136 (20.4%)		20.3	22 (5.0%)		13.9	121 (53.3%)
**BioPlex IgG** (1)	Observed S/CO	1358	N/A^b^	0	916	N/A^b^	0	442	7.3	338 (76.5%)
	Predicted S/CO		8.3	0		9.2	0		7.4	333 (75.3%)
**Trep-Sure** (4)	Observed S/CO	293	17.8	62 (21.2%)	227	18.3	36 (15.8%)	66	14.3	40 (60.6%)
	Predicted S/CO		24.6	42 (14.3%)		28.8	11 (4.8%)		34.7	5 (7.6%)

aObserved S/CO value above which 100% of specimens are confirmed positive for syphilis.

bRate of confirmation in all RPR and RPR-negative subgroups was 99.7% and 96.0%, respectively, due to four samples confirmed negative for syphilis with a screening S/CO ratio >8.0. Abbreviations. S/CO: signal-to-cutoff; RPR: rapid plasma reagin; EIA/CIA: enzyme immunoassay/Chemiluminescence immunoassay; N/A: not applicable

For the Architect TP kit, the observed optimal S/CO value providing a 100% rate of confirmation is 6.9 on low titer RPR specimens, corresponding to 70.9% of unrequired confirmation of this category. For the Trep-Sure kit, an optimal S/CO value of 17.8 applied to all RPR leads to 21% of unrequired confirmation of all samples. Because the BioPlex IgG kit provides an upper limit S/CO value of 8.0, an optimal observed S/CO value corresponding to 100% confirmation could only be established for the low-titer RPR category, corresponding to 76.5% of unrequired confirmation for this subgroup.

For the two other categories related to BioPlex IgG kit, a S/CO value could only be obtained by decreasing the unrequired confirmation rate to 99.7% (all sera) and 96.0% (RPR negative sera), due to four RPR negative samples with a S/CO index > 8.0 that were confirmed negative for syphilis. Concerning the Syphilis EIA II kit, the lack of linear correlation between S/CO index and the confirmation rate led us to not further investigate this kit.

**Fig 1:**
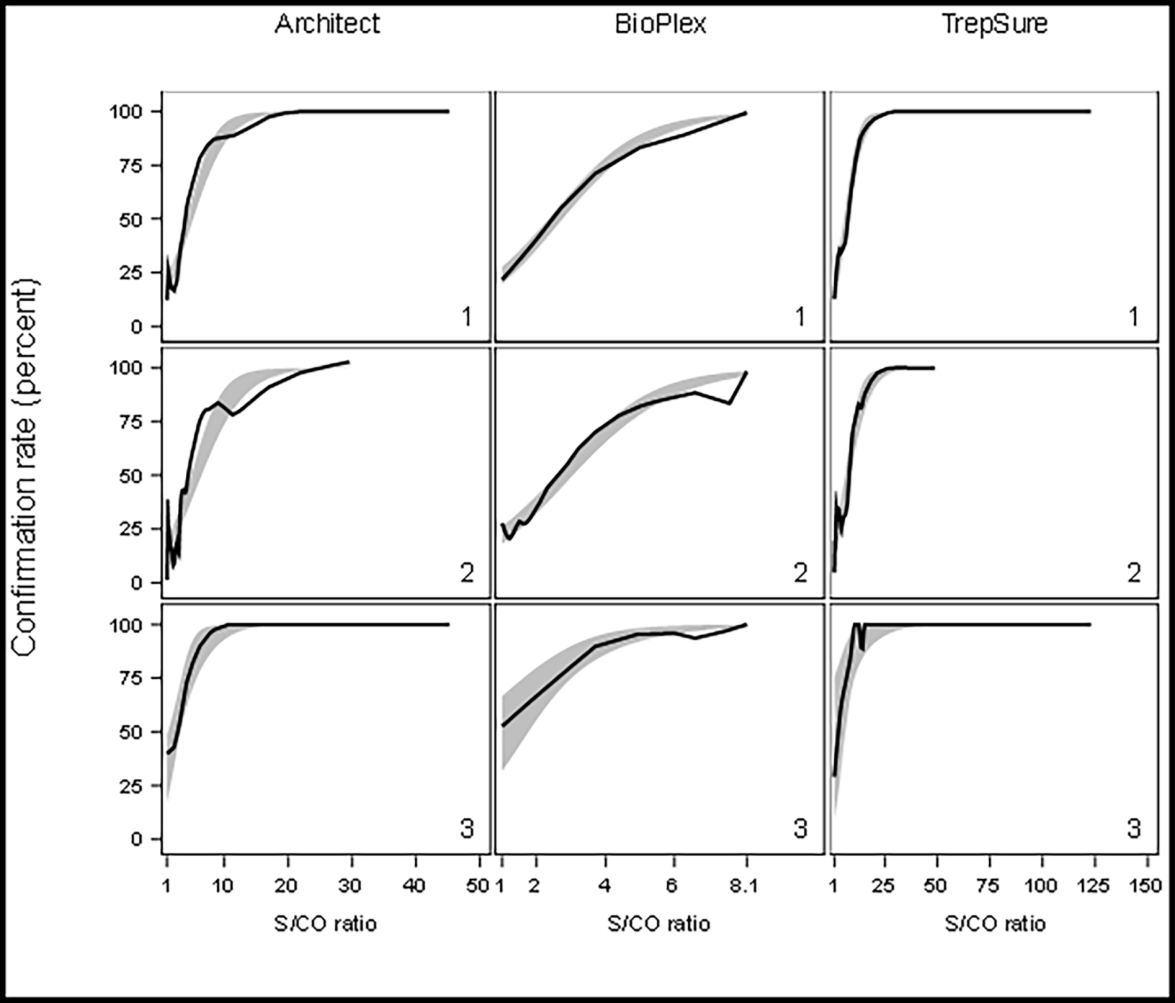
The proportion of syphilis confirmation as a function of the S/CO ratio for the Architect TP (Architect), BioPlex IgG (BioPlex) and Trep-Sure (TrepSure) screening commercial assays. **EIA/CIA reactive specimens are grouped as RPR negative and low titer RPR (1), RPR negative (2) or low titer RPR (3) results.** Grey area: modeled probability of syphilis confirmation rate as a function of S/CO values and represented the 95% confidence interval of the predicted mean value. Black lines: linear distribution of observed S/CO values. S/CO, signal-to-cutoff; RPR, rapid plasma reagin, CI, confidence interval.

A regression analysis was used to establish the optimal S/CO values (95% CI = 0.98) able to predict positive results of first-line TT tests correlating with 100% confirmation rate (Fig 1**)**.

Results obtained with the Trep-Sure, Architect TP and BioPlex IgG kits demonstrated an adequate calibration (observed syphilis confirmation rate was mainly in the 95%CI of the predicted rate). Discrimination was excellent and outstanding for the three assays (data not shown). Interestingly, the linear distribution of predicted S/CO values above which no confirmation testing would be required, superimposed our observational data. As shown Table 2, for the Architect TP and the Trep-Sure kits, the number of unrequired syphilis confirmation tests as a function of predicted S/CO ratio was higher for all sera (136 and 42 for Architect and Trep-Sure, respectively) as compared to the two subgroups (negative RPR: 22 and 11 for Architect and Trep-Sure, respectively and low-titer RPR: 121 and 5 for Architect and Trep-Sure, respectively). Because of the S/CO index upper limit of 8.0 provided by the automated BioPlex IgG system, a predictive value may only be obtained for the low-titer RPR subgroup.

Indeed, the S/CO threshold values for unnecessary confirmation (95%CI = 0.98) of the Architect TP kit for all sera was 16.4 as compared to 20.3 and 13.9 for the negative RPR sera and the low-titer RPR sera subgroups respectively. On the other hand, regression analysis performed on the Trep-Sure kit results established S/CO threshold values of 24.6, 28.8 and 34.7 for all RPR, negative RPR and low-titer RPR subgroups, respectively. For the BioPlex IgG kit, a S/CO threshold value established for the low-titer sera subgroup is 7.4. Those S/CO values corresponded to optimal unrequired confirmation rates of 20.4%, 14.3% and 75.3% for the Architect TP kit (all samples), the Trep-Sure kit (all samples) and the BioPlex IgG kit (low-titer RPR), respectively.

### Model validation of previously established predicted S/CO values

The validity of predicted S/CO thresholds was investigated using clinical specimens tested between May 2015 and February 2017. A total of 4 795 samples was tested (BioPlex IgG: 2 534, Architect: 1 921, and Trep-Sure: 340). When subjected to NTT, 3536 were negative RPR while 475, 428 and 356 presented with low RPR titer at 1, 2 and 4, respectively (data not shown). The rate of confirmation for all samples was 44%, 74% and 56% for the Architect TP, BioPlex IgG and Trep-Sure kits, respectively (data not shown). For the negative RPR subgroup, the rate of confirmation was 34%, 63% and 52% for Architect TP, BioPlex IgG and Trep-Sure, respectively (data not shown). Concerning the low titer RPR subgroup, the rate of confirmation was much higher than the other two subgroups (77%, 98% and 85% for the Architect TP, BioPlex IgG and Trep-Sure kits, respectively (data not shown). The observed S/CO values above which 100% of confirmatory testing would be unnecessary were lower or equal to the previously established predicted S/CO values, Table 3.

**Table 3:** Observeda and predicted S/CO values above which no confirmatory testing would be required: Model Validation using data from May 2015 to February 2017.

EIA/CIA Assays (number of laboratories)	All samples				RPR negative samples			Low titer RPR samples		
		*Total*, *n*	*S/CO*	*Unrequired sample confirmation*, *n (%)*	*Total*, *n*	*S/CO*	*Unrequired sample confirmation*, *n (%)*	*Total*, *n*	*S/CO*	*Unrequired sample confirmation*, *n (%)*
**Architect TP** (19)	Observed S/CO	1921	16.4	355 (18.5%)	1490	16.4	146 (9.8%)	431	11.2	248 (57.5%)
	Predicted S/CO		16.4	355 (18.5%)		20.3	101 (6.8%)		13.9	230 (53.4%)
**BioPlex IgG** (1)	Observed S/CO	2534	N/A^b^	0	1747	N/A^b^	0	787	7.3	641 (81.4%)
	Predicted S/CO		8.3	0		9.2	0		7.4	638 (81.1%)
**Trep-Sure** (3)	Observed S/CO	340	23.7	52 (15.3%)	299	23.7	36 (12.7%)	41	6.2	29 (70.7%)
	Predicted S/CO		24.6	45 (13.2%)		28.8	22 (7.4%)		34.7	2 (4.9%)

aObserved S/CO value above which 100% of specimens are confirmed positive for syphilis.

bRate of confirmation in all RPR and RPR-negative subgroups was 99.6% and 99.4%, respectively, due to ten samples confirmed negative for syphilis with a screening S/CO ratio >8.0. Abbreviations. S/CO: signal-to-cutoff; RPR: rapid plasma reagin; EIA/CIA: enzyme immunoassay/Chemiluminescence immunoassay; N/A: not applicable

The observed S/CO value for Trep-Sure (all samples) showed in Table 3 was higher (23.7) as compared to Table 2 (17.8). Indeed, amongst the 340 patients tested, three were syphilis negative and presented with a S/CO > 15.0 (18.5; 20.9 and 23.7). We also noticed a difference between the observed Trep-Sure S/CO values (6.2 in Table 3 as compared to 14.3 in Table 2) among the low titer RPR category. In this case, one of the 66 patients tested presented with a S/CO >5.5 (14.3) and was confirmed negative.

The use of an S/CO value of 16.4 with the Architect TP kit and of 24.6 for the Trep-Sure kit would obviate the need for confirmation of 18.5% and 13.2% of sera from the all RPR subgroup, respectively. For the BioPlex IgG kit, 81.1% of sera would not require confirmation when using the S/CO value of 7.4 in the low titer RPR subgroup, representing 25.2% of unnecessary confirmation of the total reactive BioPlex IgG specimens (638/2534).

## Discussion

The resurgence of syphilis infections occurring over the last decade forced many diagnostic laboratories to use rapid high throughput screening to counter such an affluence of clinical samples. With no approved NTT in Canada by the time this study was undertaken, the province of Quebec implemented a RSA. In order to anticipate the cost-effectiveness of such a RSA, we proposed to establish, for the four mainly used syphilis screening kits in Quebec, the S/CO thresholds above which no more treponemal confirmation would be required.

The large number of specimens tested reduces the putative impact of variability inter-lots or inter-testing personnel. In addition, the specimens were not submitted to freeze-thaw cycles that can deteriorate their quality. For both parts of the study (determination of S/CO and model validation), the rate of confirmation varied from 56 to 60% for TrepSure, from 56 to 44% for Architect and was 74% with BioPlex. The BioPlex displayed the highest rate of confirmed cases. This can be explained at least in part by the fact that the only laboratory using Bioplex in the province is located in Montreal where the highest rate of incidence of syphilis is observed. The incidence rates of syphilis in Montreal were 22, 26 and 38 per 100.000 population in 2014, 2015 and 2016, respectively. In the meantime, the incidence rates of syphilis outside Montreal were 6, 7 and 9 per 100.000 population in 2014, 2015 and 2016, respectively (data extracted from the Central Registry of Notifiable Diseases). The laboratories using Architect and Trep-Sure kits are well distributed in both Montreal and outside Montreal. The model validation rate exhibited a lower confirmation rate using Architect assay (44%) as compared to the S/CO obtained in the confirmatory study (56%). This change might be attributed to the increase of the number of testing laboratories, from 13 between January 2014 and April 2015 (668 tested specimens) to 19 between May 2015 and February 2017 (1921 tested specimens); the 6 additional laboratories mainly located outside of Montreal were using Architect as syphilis screening test.

No correlation was established between syphilis confirmation rates and S/CO values using the Syphilis EIA-II kit. In counterpart, a S/CO threshold value may only be obtained for the low-titer RPR sera subgroup using the BioPlex IgG kit. This result may have a significant impact on workload taking into account that the BioPlex IgG kit is used in a setting of high syphilis incidence.

Few studies investigated the correlation between the BioPlex IgG S/CO value and confirmatory results. Indeed, Fakile *et al*. [15] obtained a S/CO ratio of 4.4 that correlated at 100% with a positive TP-PA reactivity suggesting that a second TT may not be necessary. In a more recent study, Berry and Loeffelholz [7] concluded that TP-PA testing is unnecessary on specimens with S/CO ≥8 using BioPlex IgG kit. In our study, the S/CO of 7.4 correlating at 100% with a positive TP-PA reactivity concerned samples presenting with low titer RPR while 0.29% of samples confirmed negative for syphilis present with a S/CO ≥8. Even though our sample size was similar to that of Berry and Loeffelholz, the non-inclusion of sera with NT titers above 4 in our study, the difference in statistical method used to establish the S/CO thresholds and the patient population tested may account for such a difference.

Using the Architect TP kit, we evidenced that negative and low titer RPR reactive sera exhibited a S/CO value of 16.4. Dai *et al*. [8] reported that whatever the NT titers, specimens subjected to Architect present with a S/CO of 9.9 or greater leading to a TP-PA confirmation rate of 100%. Such a difference could be explained by the sample size (668 sera *versus* 319, respectively) and also by the fact that only sera less likely to represent true syphilis cases and presenting with low titers, were included in our study. The S/CO value of 16.4 was validated on a group of 1921 additional patients from 19 distinct laboratories, strengthening the usefulness of this threshold in our setting. Jonckheere *et al*. [16] tested 178 RPR negative samples using Architect at S/CO values ranged between 1 and 15. Based on TP-PA confirmatory assay, specimens with S/CO > 5.6 were considered positive and do not require further confirmation testing as compared to the higher S/CO threshold of 16.4 predicted in our study. Such a difference can be explained by the sample size (668 versus 178 samples) in addition to the S/CO range [1–15] used in each study as well as by the fact that only specimens with a low Architect value were included in Jonckheere *et al*. study.

Wong *et al*. [10] observed a good correlation between OD values and confirmatory rates with TP-PA upon studying 279 sera from a high-risk population and reactive with the Trep-Sure kit and either negative or positive by NTT. They reported that a S/CO value greater than 8.0 is associated with a confirmation rate of 99.6%. The S/CO value we obtained is quite higher than this. This may partly be due to the fact that we included only negative (S/CO = 28.8) and low-level (S/CO = 34.7) sera in our study, thereby sera that are less likely to represent true syphilis cases.

Our study has some limitations. First, we did not correlate syphilis risk factors with optimal S/CO values especially with our specific context of participating laboratories using distinct RPR and/or EIA/CIA assays. Secondly, not only the incidence of syphilis is much higher in Montreal as compared to other areas in the province but the only laboratory using BioPlex is located at Montreal. Notwithstanding such limitations, 95% of laboratories that use the Architect and Trep-Sure kits are equally distributed in the province. Indeed, we are confident that our results reflect the general population.

Based on the present results, applying such S/CO thresholds using the Architect TP, Trep-Sure or Bioplex IgG kits may improve the cost-effectiveness of the RSA in our settings by: 1) Reducing the total number of confirmatory testing (reduced number of TP-PA and/or LIA) by approximately 18.5%, 13.2% and 81.1%, respectively; 2) Decreasing the workload for the medical laboratories and the reference laboratory; 3) Improving turnaround time and therefore shortening patient intervention. Indeed, correlating S/CO values to confirmation rate for a given screening TT is easily implementable and represents a reliable way of optimizing effectiveness of syphilis serodiagnostic algorithms.
